# Biological Network Inference With GRASP: A Bayesian Network Structure Learning Method Using Adaptive Sequential Monte Carlo

**DOI:** 10.3389/fgene.2021.764020

**Published:** 2021-11-29

**Authors:** Kaixian Yu, Zihan Cui, Xin Sui, Xing Qiu, Jinfeng Zhang

**Affiliations:** ^1^ Department of Statistics, Florida State University, Tallahassee, FL, United States; ^2^ Department of Biostatistics and Computational Biology, University of Rochester, Rochester, NY, United States

**Keywords:** Bayesian network, Bayesian network structure learning, sequential Monte Carlo, adaptive sequential Monte Carlo, GRASP for BN structure learning, biological network inference

## Abstract

Bayesian networks (BNs) provide a probabilistic, graphical framework for modeling high-dimensional joint distributions with complex correlation structures. BNs have wide applications in many disciplines, including biology, social science, finance and biomedical science. Despite extensive studies in the past, network structure learning from data is still a challenging open question in BN research. In this study, we present a sequential Monte Carlo (SMC)-based three-stage approach, GRowth-based Approach with Staged Pruning (GRASP). A double filtering strategy was first used for discovering the overall skeleton of the target BN. To search for the optimal network structures we designed an adaptive SMC (adSMC) algorithm to increase the quality and diversity of sampled networks which were further improved by a third stage to reclaim edges missed in the skeleton discovery step. GRASP gave very satisfactory results when tested on benchmark networks. Finally, BN structure learning using multiple types of genomics data illustrates GRASP’s potential in discovering novel biological relationships in integrative genomic studies.

## Introduction

A Bayesian network (BN) is a graphical representation of the joint probability distribution of a set of variables (called nodes in the graph). BNs have been widely used in various fields, such as computational biology (Friedman, Linial, Nachman and Pe’er 2000; Raval, Ghahramani and Wild 2002; Vignes, et al., 2011), document classification (Denoyer and Gallinari 2004), and decision support system (Kristensen and Rasmussen 2002). A BN encodes conditional dependencies and independencies (CDIs) among variables into a directed acyclic graph (DAG). And this DAG is called the structure of a BN. When the structure of a BN is given, the parameters that quantify the CDIs can be estimated from observed data. If neither the structure nor parameters are given, they can be inferred from observed data. In this study, we will be focusing on the structure estimation of a BN and its application in learning biological networks using heterogeneous genomics data.

The technical difficulties of structure learning are mainly due to the super-exponential cardinality of the DAG spaces, which are also quite rugged for most commonly used score functions. Estimating the structure exactly is an NP-hard problem (Cooper 1990; Koller and Friedman 2009). There have been many inexact and heuristic methods proposed in the past 2 decades (Zhang, Li, Zhou and Wei 2013; Adabor, Acquaah-Mensah and Oduro 2015; Larjo and Lahdesmaki 2015; Amirkhani, Rahmati, Lucas and Hommersom 2017; Franzin, Sambo and Di Camillo 2017; Han, Zhang, Homayouni and Karmaus 2017; Ferreira-Santos, Monteiro-Soares and Rodrigues 2018; Jabbari, Visweswaran and Cooper 2018; Li and Guo 2018; Tang, Wang, Nguyen and Altintas 2019; Zhang, Wang, Duan and Sun 2019; Zhang, Rodrigues, Narain and Akmaev 2020; Liu, Gao, Wang and Ru 2021). The strategy of these methods can be classified mainly into three categories: constraint-based, score-based, and hybrid, which combines both constraint-based and score-based approaches.

A constraint-based method utilizes the conditional dependency test to identify the conditional dependencies and independencies among all the nodes (Campos 1998; de Campos and Huete 2000; Margaritis 2003; Tsamardinos, Aliferis and Statnikov 2003; Yaramakala and Margaritis 2005; Aliferis, Statnikov, Tsamardinos, Mani and Koutsoukos 2010; Liu, et al., 2021). A major disadvantage of such a method is that a large number of tests have to be conducted; therefore, an appropriate method to adjust the *p*-values obtained from all the tests have to be applied. The fact that not all the tests are mutually independent further complicates the *p*-value adjustment. Another issue is that the goodness-of-fit of the obtained network is usually not considered in such an approach; therefore, the estimated BN may not fit the observed data well.

A score-based method uses a score function to evaluate the structures of BNs on observed data (Larrañaga, Poza, Yurramendi, Murga and Kuijpers 1996; Friedman, Nachman and Peér 1999; Gámez, Mateo and Puerta 2011). A searching algorithm is employed to search the best BN (with the highest score) with respect to certain score function. Various Bayesian and non-Bayesian score functions have been proposed in the past. As exact search is not feasible, over the past 2 decades, various heuristic searching methods, such as hill climbing, tabu search, and simulated annealing were proposed to search for the optimal BN structures. The problem with score-based method is that the searching space is often very large and complicated; therefore, the searching algorithm either will take too much time to find the optimum or be trapped in local optima. Many efforts have been made to overcome this challenging issue, such as searching using an ordered DAG space to reduce the searching space (Teyssier and Koller 2012). In the ordered DAG space, the nodes are given an order such that edges will only be searched from higher orders to lower orders. The practical issue is that determining the orders and finding the optimal structure is equally difficult. More recently, various penalty-based methods were proposed to estimate the structures for Gaussian BN (GBN) (Fu and Zhou 2013; Huang, et al., 2013; Xiang and Kim 2013). These methods have been shown to be quite efficient for GBN structure learning and are able to handle structure learning and parameter estimation simultaneously; however, these methods are quite restrictive: the joint distributions must approximately follow a multivariate Gaussian distribution and dependencies among nodes are assumed to be linear.

Hybrid methods which combine a constraint-based method and a score-based method were proposed to combine the advantages of both methods (Tsamardinos, Brown and Aliferis 2006). Such methods often contain two stages: first pruning the searching space by a constraint-based methods, then searching using a score function over the much smaller pruned space. In the pruning stage, the goal is to identify the so-called skeleton of the network, which is the undirected graph of the target DAG. Later in the second stage, the direction of each edge will be determined by optimizing the score function. In a hybrid method, it is important that the first stage identifies as many true undirected edges as possible, since only the identified undirected edges will be considered in the second stage.

In this study, we developed a novel BN structure learning method named GRASP (GRowth-based Approach with Staged Pruning). It is a three-stage method: in stage one, we used a double filtering method to discover a cover of the true skeleton. Unlike the traditional constraint-based methods, which try to obtain the true skeleton exactly, our method only estimates a super set of the undirected edges and it only conditions on at most one node other than the pair of nodes being tested, which dramatically reduces the number of observations needed to make the test results robust. In stage two, we designed an adaptive sequential Monte Carlo (adSMC) (Liu and Chen 1998; Liu 2008) approach to search for a BN structure with optimal score based on constructed skeleton. SMC has been successfully adopted to solve optimization problems in the past (Grassberger 1997; Liang et al., 2002; Zhang and Liu 2002; Zhang et al., 2003; Zhang et al., 2004; Zhang and Liu 2006; Zhang et al., 2007; Zhang et al., 2009). Compared to most greedy searching methods, SMC is less likely to be trapped in local optima. Another advantage of SMC is that it can be run in parallel for each SMC sample, making it suitable for distributed or GPU-based implementations. To further increase the efficiency of the sampling, an adaptive SMC strategy was used to generate higher scored networks. After these two stages, we enhanced the traditional two-stage approach with a third stage which adds possible missed edges back into the network using Random Order Hill Climbing (ROHC).

## Methods and Data

### GRASP: GRowth-Based Approach With Staged Pruning

GRASP is a three-stage algorithm for learning the structure of a BN. In the first (pruning) stage, we designed a Double Filtering (DF) method to find the cover of the skeleton of the BN, where the skeleton of a BN is defined as the BN structure after removing the direction of all the edges, and the cover is defined as a superset of undirected edges containing all the edges of the skeleton. In the second (structure searching) stage, we developed an adaptive sequential Monte Carlo (adSMC) method to search the BN structure on the undirected network found in the first stage based on Bayesian information criterion (BIC) score. To reclaim the potentially missed edges, we designed a Random Order Hill Climbing (ROHC) method as the third stage.

### First Stage: Double Filtering (DF) Method to Infer the Skeleton

The first stage, namely Double Filtering (DF) method, contains two filtering processes. The first filtering was done by unconditioned tests, filtering out the nodes that are not ancestors or descendants of a given node $X_{i}$ . The second filtering was built on conditioned tests, further filtering out the nodes that are not direct neighbors (parents or children) of $X_{i}$ .

Suppose we have p nodes. For a given node $X_{i},$ let $\text{nbr}\left(X_{i}\right)$ be the set of nodes that have an undirected edge with $X_{i}$ . Initialize $\text{nbr}\left(X_{i}\right)=\emptyset$ the empty set for all i . The procedure of the DF method is as follows:

1. *First filtering*. For each pair of nodes ( $X_{i}$ and $X_{j}$ , i≠j ), record the *p*-value of their Mutual Information (MI) test as $p_{\text{ij}}$ . If $p_{\text{ij}}<\alpha$ , α being a predetermined significance level for the test, add $X_{j}$ into $nbr\left(X_{i}\right)\;$ and $X_{i}$ into $nbr\left(X_{j}\right)$ . The MI test is also used by other BN structure learning methods (Campos 1998). Let $N_{i}$ be the number of elements in $\text{nbr}\left(X_{i}\right)$ after first filtering. Sort $\text{nbr}\left(X_{i}\right)$ using their p -values in ascending order and denote these nodes as $X_{1}^{\left(i\right)},\;X_{2}^{\left(i\right)},\;\ldots ,X_{N_{i}}^{\left(i\right)}$ .
2. *Second filtering.* For every node $X_{i}$ , initialize the set of its final neighbors ${\text{nbr}}^{C}\left(X_{i}\right)=\text{nbr}\left(X_{i}\right)$ . Loop over its elements in the order of $X_{1}^{\left(i\right)},\;X_{2}^{\left(i\right)},\;\ldots ,X_{N_{i}}^{\left(i\right)}$ .
  (a). For every element $X_{j}^{\left(i\right)}$ , find $\text{nbr}\left(X_{i}\right)\cap \text{nbr}\left(X_{j}^{\left(i\right)}\right)$ , the intersection of the neighbors of $X_{i}$ and $X_{j}^{\left(i\right)}$ .
  (b). For every element $X_{k}^{\left(i,j\right)}$ in the set of intersection, perform a conditional dependency test for $X_{i}$ and $X_{k}^{\left(i,j\right)}$ , given $X_{j}^{\left(i\right)}.$ If the p -value >α , remove $X_{j}^{\left(i\right)}$ from ${\text{nbr}}^{S}\left(X_{i}\right)$ .

After applying the DF method on all p nodes, the collection of ${\text{nbr}}^{C}\left(X_{i}\right),\;i=1,2,...,p$ gives us the skeleton of BN.

### Second Stage: Structure Searching

In the pruned space, we designed an adaptive sequential Monte Carlo (adSMC) method to search the structure of the Bayesian network. In a traditional sequential Monte Carlo, the random variable of all p nodes (or features) $X\in {\mathbb{R}}^{p}$ is decomposed into M blocks $\left(x_{1},\;x_{2},\;\ldots ,\;x_{M}\right)$ each with $p_{i}$ features $x_{i}\in {\mathbb{R}}^{p_{i}}$ and $\sum_{i=1}^{M}p_{i}=p$ , and the decomposition is predefined and fixed throughout the whole sampling procedure. One usually samples $\;x_{1}$ at first, then $x_{2}$ , and so on. However, the sequence each variable is sampled (namely sampling sequence in this study) based on any prior decomposition may not be the most efficient one. The optimal sampling sequence may need to be decided dynamically. For example, when $x_{1},\;x_{2},\ldots ,\;x_{m-1}$ have been sampled for some 0<m≤M , the conditional distribution $f\left(\;x_{m}|x_{1},\;x_{2},\ldots ,\;x_{m-1}\right)$ may have a small set of candidate decompositions (to satisfy the acyclic condition) which limits the diversity of the SMC samples. Therefore, we designed our sampling block $x_{m}$ conditioning on the current sampled structure $x_{1},\;x_{2},\ldots ,\;x_{m-1}$ to increase the diversity and quality of obtained samples (see Supplementary Figure S1 in Supplementary Materials for an example).

**FIGURE 1:**
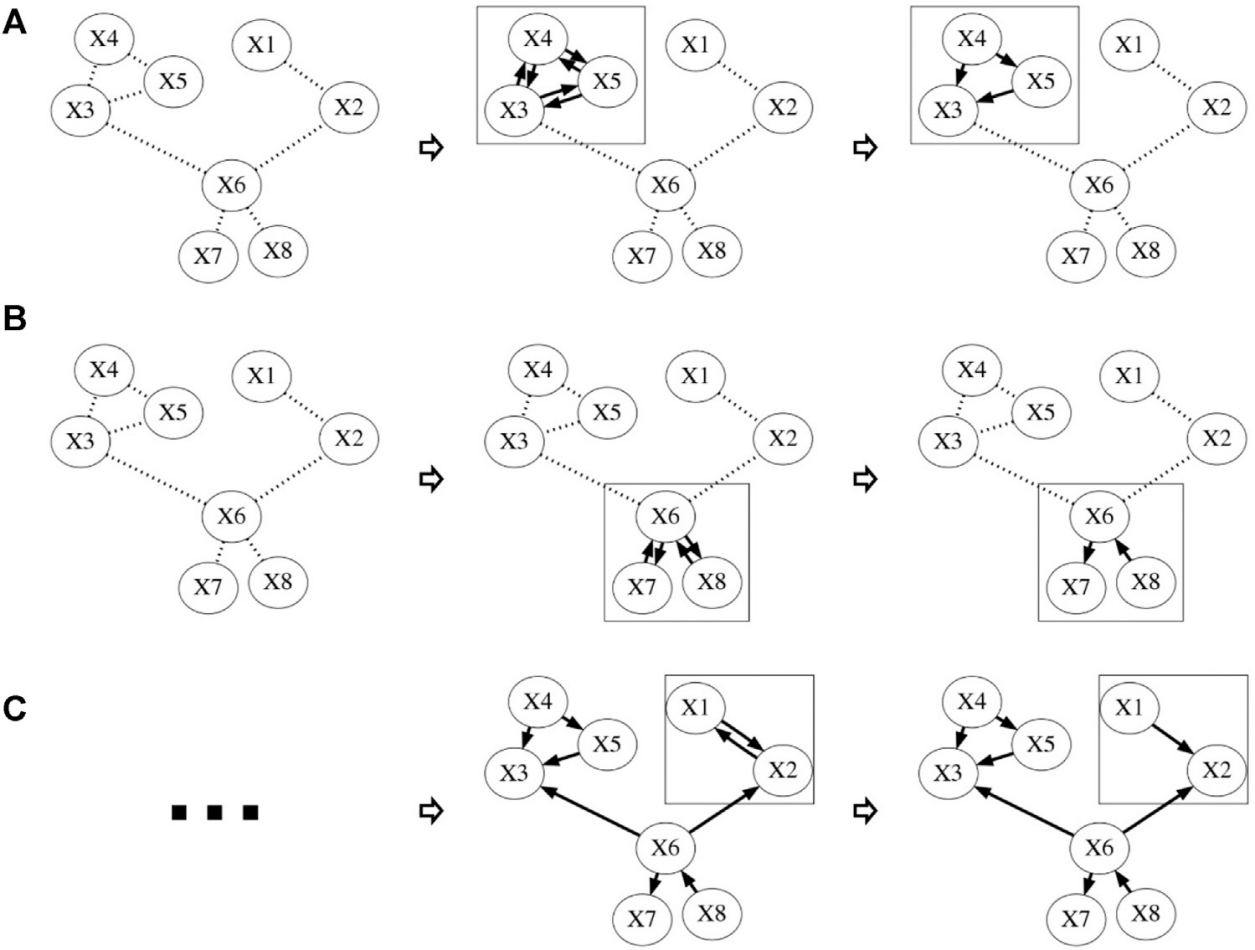
Structure discovering procedure.

For each SMC sample, we start with all possible fully connected triplets (three nodes connected by three undirected edges) discovered in the first stage. We sample one such triplet having the least outside connection, e.g., the one having the least undirected edges connected to its nodes (Figure 1A). These triplets are likely to be restricted to certain configuration by the sampled structure; therefore, to sample them earlier allows more variety in their configurations. When all fully connected triplets are sampled, partially connected triplets (two undirected edges among three nodes) are considered (Figure 1B). Lastly, we consider pairs (the remaining undirected edges, Figure 1C). For partially connected triplets and pairs, the configurations with the least outside connections are sampled first.

The probabilities of possible configurations of triplets and pairs are proportional to their BIC (Bayesian Information Criterion) score defined as $\text{BIC}=\text{ln}\left(n\right)\cdot k-2\cdot \text{ln}\left(\hat{L}\right)$ , where  n represents sample size, k represents number of parameters of the partial BN model, and $\hat{L}=p\left(x|\hat{\theta },\text{Model}\right)$ is the maximized value of the likelihood function of the model, with estimated parameters $\hat{\theta }$ from observed data  x . The probability is set to be 0 if certain configuration fails to satisfy the acyclicity condition. In summary, for triplets (pairs), we calculate the BIC for all possible configurations of this triplet (pair) and sample one configuration with probability

$$P\left(\text{configuration}\;i\right)\propto \{\begin{array}{cc} \exp\left(\frac{{\text{BIC}}_{i}}{T}\right), & \text{ if }\;\text{configuration}\;i\;\text{does}\;\text{not}\;\text{result}\;\text{in}\;\text{a}\;\text{loop}\\ 0, & \text{if }\;\text{configuration}\;i\;\text{result}\;\text{in}\;\text{a}\;\text{loop} \end{array},$$ (1)

Where exp(⋅) is the exponential function, T is temperature controlling how greedy we want the searching to be, and ∝ means proportional to.

The main algorithm used in the second step is as follows.

Step 1 Sample one fully connected triplet $\left\{X_{I},X_{J},X_{K}\right\}$ with the least outside connection; Choose a configuration between these three nodes with probability described in Eq. 1; Then remove the connection between $X_{I}$ , $X_{J}$ and $X_{K}$ from the skeleton.

Step 2 Repeat step 1 until all fully connected triplets are sampled.

Step 3 Sample one partially connected triplet $\left\{X_{I},X_{J},X_{K}\right\}$ with the least outside connection. Choose a configuration between these three nodes with probability described in Eq. 1. Then remove the connection between $X_{I}$ , $X_{J}$ and $X_{K}$ (if applicable) from the skeleton.

Step 4 Repeat step 3 until all partially connected triplets are sampled.

Step 5 Sample one pair $\left\{X_{I},X_{J}\right\}$ with the least outside connection. Choose a configuration between them (either $\;X_{I}\to X_{J}\;$ or $\;X_{I}\leftarrow X_{J}$ ) with probability described in Eq. 1. Then remove the connection between $X_{I}$ and $X_{J}$ from the skeleton.

Step 6 Repeat step 5 until all pairs are sampled and no more unsampled edges in the skeleton.

Since each SMC sample is generated independently, we can run our algorithm in parallel on multiple CPUs/GPU cores to speed up the sampling process.

### Third Stage: Reclaiming Missed Edges

We mentioned earlier that one disadvantage of the traditional two-stage method was that the edges missed in the first stage will never be recovered. Therefore, in the third stage we designed a Random Order Hill Climbing (ROHC) method to identify the possible missed edges and refine the network. The general idea is described as follows:

1. Generate a permutation of 1,2,…, p for each network sampled by adSMC in stage 2, suppose $m_{1},\ldots ,\;m_{p}$ is such a permutation.
2. For every node $X_{i}$ , iterate *j* from $m_{1}$ through $m_{p}$ . If $X_{i}\leftarrow X_{j}$ does not create loop and result in an increasing in BIC, we add edge $X_{i}\leftarrow X_{j}$ .
3. Repeat (2) until there is no possible edge to add or the searching limit is reached.

One could also view this stage as a further ascent to the local optima to ensure we achieve the best possible BIC score.

In `general, generating more SMC samples gives a higher chance to reach the optimum. However, more samples also require more computation time; therefore, a balance between running time and sample sizes must be made. In most of our simulation study and practical problems, we found that around 20,000 samples were often good enough for finding a network with a satisfactory BIC score.

### Performance Evaluation

To measure the effectiveness of edge screening methods, we employed the precision, recall and f-score measurements. Precision is defined as TP/(TP + FP), recall is defined as TP/(TP + FN), and f-score is the harmonic mean of precision and recall, 2 (precision × × recall)/(precision + recall), where TP means true positive (number of true undirected edges identified), FP false positive (number of non-edges identified as undirected edges), and FN false negative (number of undirected edges not identified).

In our study, recall measures the percentage of true edges (irrespective of their directions) identified; therefore, it is the most important measurement in edge screening stage, since as we discussed earlier, any missed edges in stage one may never be reclaimed in a traditional two stage approach. Besides the recall, f-score is also important since it measures a balanced performance in terms of both precision and recall. It is obvious that if we propose all possible edges, we will always identify all true edges, but that will not do any pruning to the searching space. Thus, a high f-score is desired for a decent edge screening strategy.

We used Bayesian Information Criterion (BIC) as the score function in both second stage and third stage. BIC has the score-equivalent property (Appendix definition 10), which can reduce the searching space, since if we could find one network in the equivalent class, we found the true network. And the consistency property of BIC score guarantees that the true network has the highest score asymptotically.

### Benchmark Networks

The networks used to generate simulated data (Table 1) are from actual decision making processes of a wide range of real applications including risk management, tech support, and disease diagnosis. All networks are obtained from Bayesian Network Repository maintained by M. Scutari http://www.bnlearn.com/bnrepository/.

**TABLE 1:** Bayesian networks used in the simulation study.

Name	# of nodes	# of edges	# of parameters	Max in-degree
Alarm	37	46	509	4
Andes	223	338	1,157	6
Child	20	25	230	2
Hailfinder	56	66	2,656	4
Hepar2	70	1,236	1,453	6
Insurance	27	52	984	3
Win95pts	76	112	574	7

We randomly generated data with 1,000, 2,000, and 5,000 observations, and we generated 10 datasets for each size of observations. All results reported in this section are based on averages of 10 datasets. Observation size in this article refers to the number of data points, and shall not be confused with number of sequential Monte Carlo samples. The datasets were generated using R package *bnlearn* (Scutari 2009; Nagarajan, Scutari and Lèbre 2013).

### Real Data

#### Flow Cytometry Dataset

In the flow cytometry dataset (Sachs, Perez, Pe’er, Lauffenburger and Nolan 2005), there are 11 proteins and phospholipid components of the signaling network. The original data was collected from 7,466 cells, containing continuous variables. Sachs et al. suggested to get rid of the potential outliers by removing data that are three standard deviations away from any attribute. Thus the data we are analyzing contains 6,814 observations. We discretized each variable into three categories, practically stands for high/medium/low, with each category containing 33% of the data.

#### Genomics and Epigenomics Data From the Cancer Genome Atlas (TCGA)

We used several different types of data obtained from TCGA: RNA-seq, protein expression, DNA methylation, and microRNA-seq, which have been used in our previous studies (Stewart, Luks, Roycik, Sang and Zhang 2013; Li, et al., 2017; Shi, et al., 2017; Li, et al., 2020). These data can be freely downloaded from TCGA data portal (https://portal.gdc.cancer.gov/), which has detailed description on each of the data types.

## Results

### Edge Screening

The principal of the edge screening stage is pruning the searching space as much as possible while the remaining edges in the pruned space still possess as many true edges as possible. We compare our method to five other methods including max-min parent-child (mmpc) (Tsamardinos, et al., 2006), grow-shrink (gs) (Margaritis 2003), incremental association (iamb) (Tsamardinos, et al., 2003), fast iamb, and inter iamb (Yaramakala, et al., 2005). For all methods, we fixed the significance level (α) to 0.01.

**FIGURE 2:**
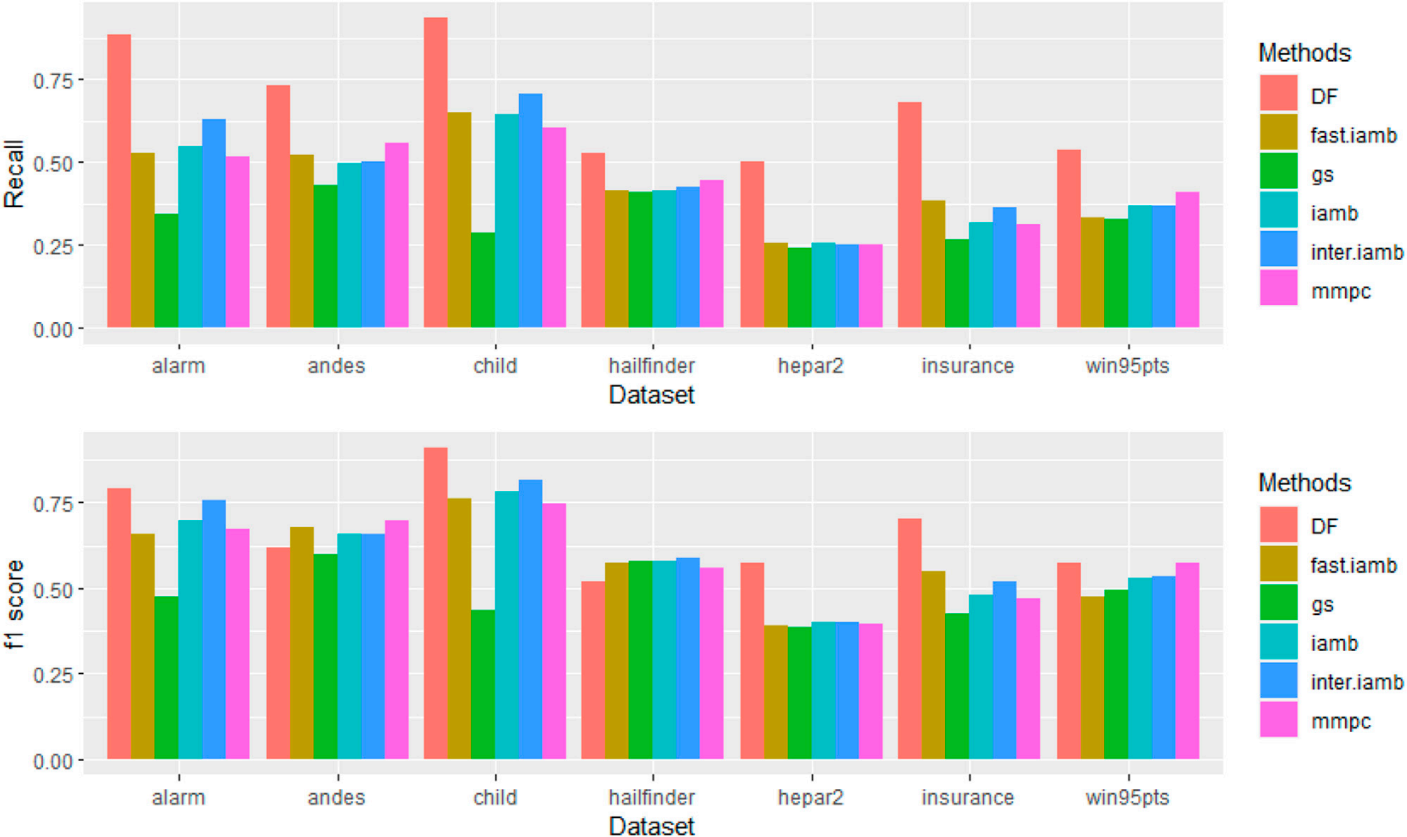
Recall and f1 score of different methods with observation size 1,000. One can see that DF generally has higher recalls with higher or comparable F1-scores for the same network.

The simulation study results (Figure 2 and Figure S2) showed that our double filtering (DF) method was able to identify the most edges (highest recall) for each of the observation size we tested. In some cases we observed that with even 1,000 observations, our method achieved a higher recall than the other methods using 5,000 observations and the f-scores are still comparable (e.g., Alarm, Hepar2 and etc.). For some networks (Child, Insurance), not only the recalls were higher but also the f-scores were higher for DF. The results confirmed that DF identifies true edges more accurately than other methods and it often requires fewer observations. Higher recall is desired in the first stage (the edge screening stage) since any missed edges will not be sampled in the second stage.

#### Effect of Temperature

The temperature parameter in SMC has the same effect as that in MCMC (Markov Chain Monte Carlo) simulations. A lower temperature will cause searching to become greedier, and higher temperatures make it less greedy. When T→0 the searching procedure becomes a local greedy search. On the other hand, when T→∞ , the configuration is sampled uniformly. The optimal temperature is usually a value in between. In this simulation study, we fixed SMC sample size to 20,000, and rounds of ROHC to 5. The temperature was set to between $10^{-7}$ and $\;10^{-1}$ , increased by 10-time each time (Figure S3). The performance is shown in the relative scale (BIC of true network/BIC of the learned network), where higher ratio means higher BIC score; thus, better network structure. Lower temperature in most cases gave a lower score, as well as the higher temperature, consistent with what we would expect. Most of the optimal scores happened around T = 0.001 or 0.01. We can also see that the optimal temperature does not depend on the observation sizes, since the optimal temperatures are the same across the three different observation sizes. Another observation we had was that the optimal temperatures do not change much when the number of variables (nodes) changes. From figure S3 we can see that for Andes (with 223 nodes) and child (20 nodes), the optimal temperature is both around 0.01 and 0.001.

#### Effect of Adaptive SMC

To show the improvement of using adaptive SMC, we compared the BICs of 20,000 SMC samples between the adSMC and traditional SMC (Figure S4). In the traditional SMC, we designed the sampling block in the order of fully connected triplets, partially connected triplets and pairs, and started from least outside connected ones. Clearly, the adSMC generates higher scored networks in general.

#### Effect of the Edge Reclaiming Step

We discussed earlier that there could be some true edges missed in the first stage due to the test power and limited data. Here we will show that Random Order Hill Climbing (ROHC) indeed improves the learned BN structure in stage 2. We used *alarm* and *win95pts* networks to illustrate the improvement made by ROHC (Figure S5). They both had significance level cut-off of 0.01, temperature 0.001, and 20,000 SMC samples. As we can see, the improvements were substantial, demonstrating that it is necessary to have the third stage to further refine the learned network. However, one should notice that the complexity level of ROHC is approximately $O\left(N^{2}\right)$ ; therefore, in a typical network with hundreds of nodes only 1 or 2 rounds of ROHC are affordable.

#### Performance on Benchmark Networks

**FIGURE 3:**
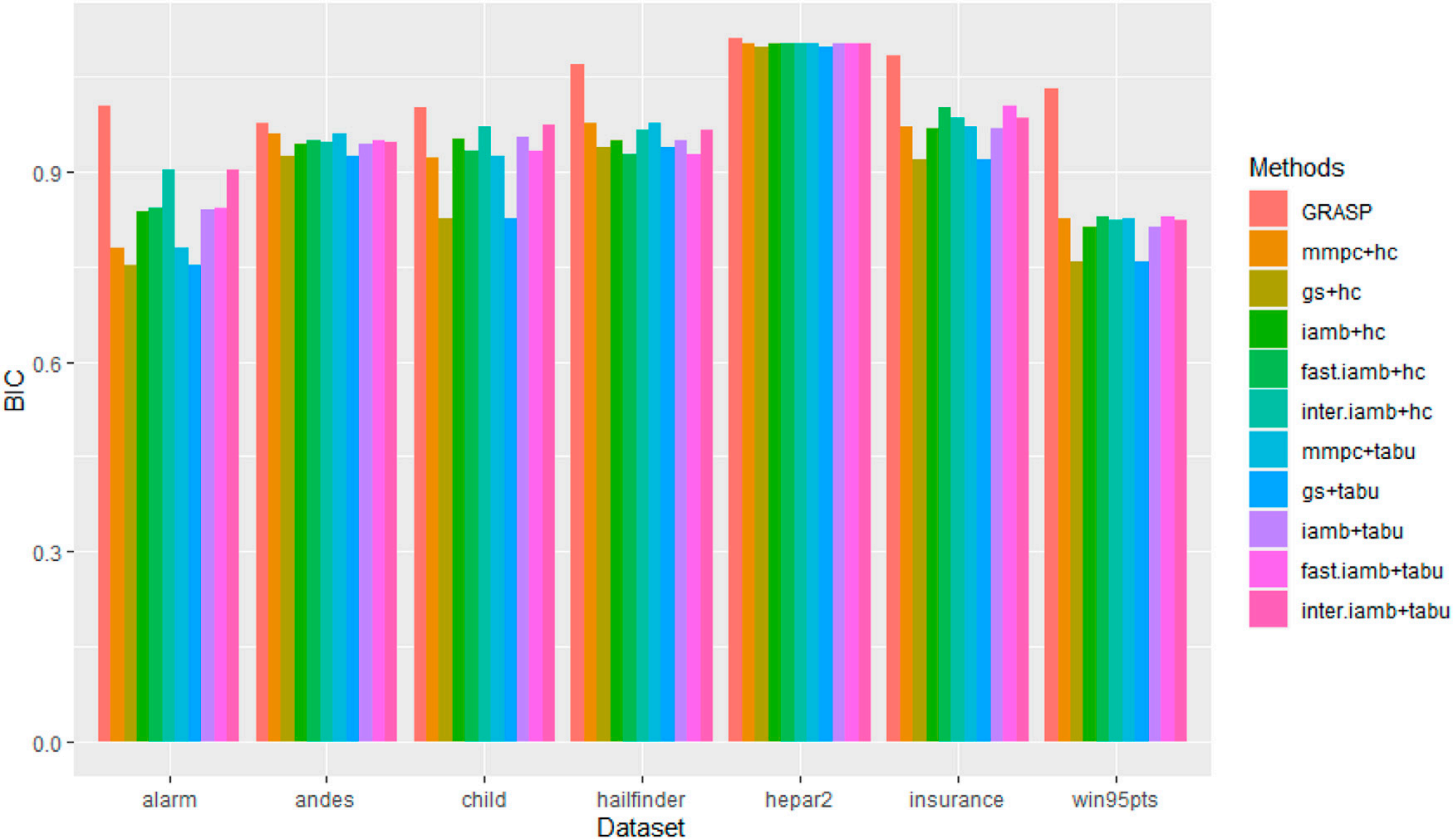
BIC scores of all methods on seven benchmark networks with observation size 1,000. GRASP has higher BIC scores for all the benchmark networks.

We evaluated the overall performance of our method and the general two stage methods (five edge screening methods, gs, mmpc, iamb, fast.iamb, and inter.iamb combined with two optimization methods, Hill climbing and tabu search) on seven benchmark networks. The results are shown in Figure 3 and Supplementary Figure S6 (Supplementary Material). For three different observation sizes, our method outperformed all the general two-stage methods on almost all benchmark networks except on the hepar2 network where all methods achieved similar scores, which are very close to the BIC of the true network.

#### Performance on the Flow Cytometry Data

**FIGURE 4:**
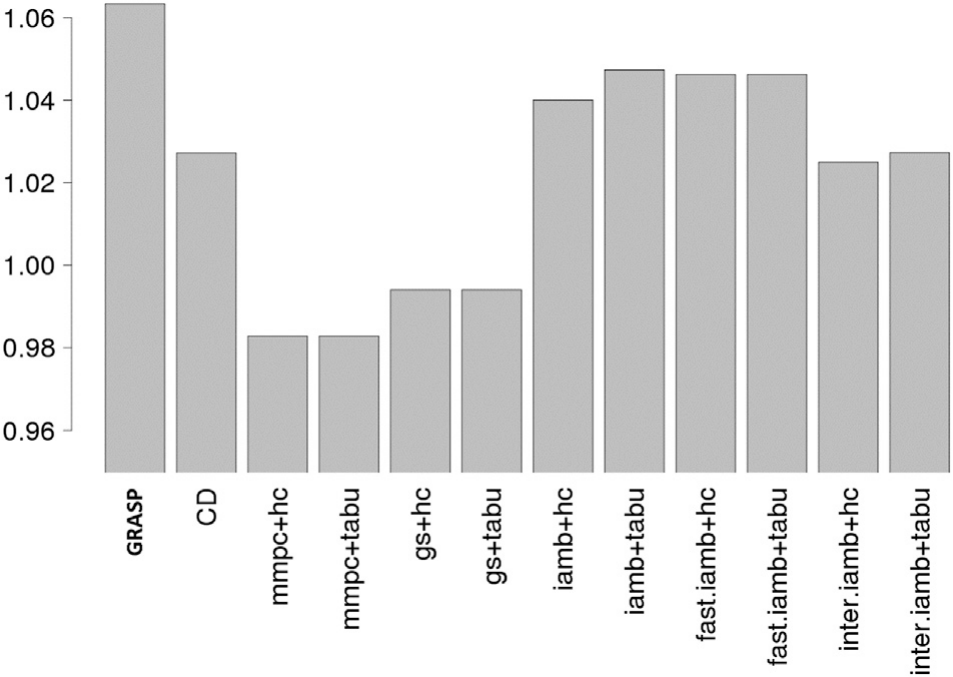
BIC scores for the flow cytometry data, comparing 12 methods, and GRASP has the highest BIC score. The y-axis value is the ratio of the BIC score of the sampled network and the true network. It is possible that a sampled network has even higher BIC score than the true network, hence the value can be higher than 1.

We first compared our method to the general 2-stage methods and the CD method (Fu, et al., 2013) on the flow cytometry data. GRASP achieved the highest BIC score (Figure 4), which is consistent with the simulation study.

#### An Integrative Genomic Study Using TCGA Data

**FIGURE 5:**
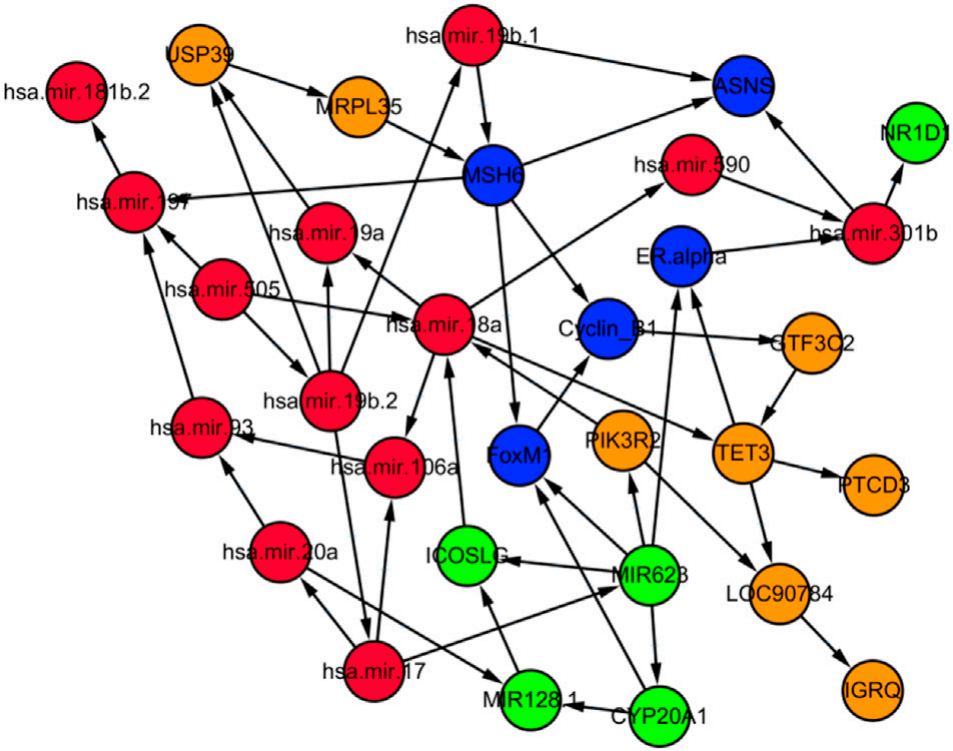
The BN structure learned by GRASP using multiple different genomic features which are highly correlated with the expression of LOC90784. Orange nodes: mRNA transcripts; Red nodes: microRNAs; Blue nodes: protein expressions; Green nodes: DNA methylations.

An advantage of BN models is that they can handle heterogeneous data well. In this section, we will test our method using a heterogeneous genomics dataset from TCGA through learning network structures that may shed light on real biological problems. In a previous study of ours (Stewart, et al., 2013), we have identified a long non-coding RNA, LOC90784, which is strongly associated with breast cancer health disparity between African American and Caucasian American breast cancer patients. However, literature search resulted in no information about it since it had not been studied by any researchers in the past. Using several different types of genomics data, we applied GRASP to perform an integrative study to build a Bayesian network with different genomics features to shed some light on the function of this transcript. All the data were first discretized into a small number of categories, usually 2–4. We first used RNA-seq data to identify transcripts highly correlated with LOC90784. This gave us eight transcripts with absolute value of correlation coefficient greater than 0.27. We then found other genomic features, including microRNAs, DNA methylations and protein expressions that are highly correlated with these transcripts, which gave us 13 microRNAs, 5 DNA methylation regions (aggregated around genes) and five proteins. Using the samples with all the above measurements, we inferred the BN structure for these genomics features as shown in Figure 5. As a comparison, bnlearn, a R package for BN structure learning, gave a network without LOC90784 (Supplementary Figure S7). Figure 5 showed rather complex relationships among all these genomic features. A thorough investigation of this network is beyond the scope of this work. However, some literature search on the nodes around LOC90784 provided interesting hypotheses, which could be followed up with experiments. Specifically, TET3, an upstream gene, was found to inhibit TGF-β1-induced epithelial-mesenchymal transition in ovarian cancer cells (Ye, et al., 2016). High frequency of PIK3R2 mutations in endometrial cancer was found to be related to the regulation of protein stability of PTEN (Cheung, et al., 2011), which is a well-known cancer related gene. There are not a lot of published studies on IRGQ. From the Human Protein Atlas database (https://www.proteinatlas.org/ENSG00000167378-IRGQ/pathology) we found that this gene is a prognostic biomarker and significant for survival for several cancer types including pancreatic cancer, renal cancer, cervical cancer and liver cancer. It would be interesting to see how perturbations of TET3, PIK3R2, such as knockdown/knockout experiments, affect LOC90784 and how perturbation of LOC90784 affects IRGQ. These hypotheses demonstrated the potential of GRASP for discovering new biology through integrative genomic studies.

## Discussion

In this study, we developed a three-stage Bayesian network structure learning method, GRASP. The first stage is an edge screening method, Double Filtering, which recovers a super set of true edges and proposes as few edges as possible. The second stage is an adaptive SMC (adSMC) approach to optimize a score function (BIC in this study) that measures the fitness of a BN structure to the given data. To reclaim the possible missed edges from the first two stages, we developed a random order hill climbing method (ROHC) to recover the missed edges as the last stage. The principal of double filtering is quite different from the well-known mmpc method or other similar constraint-based methods, where the algorithm is trying to identify the skeleton of the BN (undirected true edges). Double filtering method focuses on identifying a set of undirected edges that contains all the true edges, at the same time tries to propose as few edges as possible. The advantage of mmpc is that given enough observations it identifies the true network skeleton; however, it may not be feasible when the observations are limited since mmpc conducts conditional dependency test conditioning on all previously identified dependent (connected) nodes, and it requires more observations when the number of conditioned nodes increases. On the other hand, double filtering only conditions on one node at a time, so the required observation size can be much smaller.

The adSMC approach in structure sampling stage can find better BN structure than greedy searching algorithms or traditional SMC. The algorithm takes into account the currently sampled partial BN structures to make more informed decisions on the sampling of new edges. In addition, adSMC sampling is completely parallelizable, and multiple CPUs/GPU implementations will likely further improve the computational efficiency substantially.

Although in this study we focused on categorical variables (nodes) with multinomial distribution, one may extend our approach to other types of variables including Gaussian ones, as long as all nodes have the same distribution and the local conditional distribution can be estimated. Imposing distributions that are easier to be estimated on the nodes will in general make the searching more efficient. Practically, it is not an easy task to find appropriate distribution for all nodes. For BNs with mixed node types, where nodes do not necessarily have the same distribution, our method could handle them indirectly by discretizing the observations making each node distributed as multinomial distribution.

The application of GRASP on heterogeneous genomics data showed its potential to infer complex biological networks, which may shed light on the functions of unknown genes or epigenetic features. The learned structures of BN also provide guidance on formulating specific hypotheses that can be tested experimentally.

## Data Availability

Publicly available datasets were analyzed in this study. Benchmark Bayesian networks can be found at: https://www.bnlearn.com/bnrepository/. Genomics data analyzed can be found at: https://portal.gdc.cancer.gov/.
