# Supplementary material for: Biological Network Inference With GRASP: A Bayesian Network Structure Learning Method Using Adaptive Sequential Monte Carlo
Source: Front Genet. 2021 Nov 29;12:764020. doi: 10.3389/fgene.2021.764020 (PMC8668238; doi:10.3389/fgene.2021.764020)
Supplement: Supplementary file 1 [file DataSheet1.docx]

**Supplementary File for: Biological network inference with GRASP: a Bayesian network structure learning method using adaptive sequential Monte Carlo**

Kaixian Yu^1,*^, Zihan Cui^1^, Xin Sui^1^, Xing Qiu^2^ and Jinfeng Zhang^1,*^

^1^Department of Statistics, Florida State University, Tallahassee, FL, 32306

^2^Department of Biostatistics and Computational Biology, University of Rochester, Rochester, NY 14624, USA

**Appendix**

**Preliminary of Bayesian Network**

Let us denote the set of $p$ variables (nodes) as $\boldsymbol{X}=\{X_{1},\ldots,X_{p}\}$, and the set of edges as $E=\{X_{i}\to X_{j}\}$ where $X_{i}\to X_{j}$ are directed edges in the graph. ­$X_{i}$ is called a parent of $X_{j}$ and $X_{j}$ a child of $X_{i}$. Thus a graph can be represented as $G(\boldsymbol{X},\boldsymbol{E})$.

**Definition 1:** For a set of nodes $X_{i_{1}},\ldots,X_{i_{k}}\in\boldsymbol{X}$ if the edges $X_{i_{1}}\to X_{i_{2}}\to\ldots\to$ $X_{i_{k-1}}\to X_{i_{k}}\in\boldsymbol{E}$, then we say $X_{i_{1}},\ldots,X_{i_{k}}\in\boldsymbol{X}$ forms a directed path between $X_{i_{1}}$ and $X_{i_{k}}$. If $X_{i_{1}}=X_{i_{k}}$, then this directed path is called a cycle.

**Definition 2:** A directed acyclic graph (DAG) is a graph $G(X,E)$ such that all edges in *E* are directed and there is no cycles in *G*.

We denote *P(****X****)* as a joint probability distribution over the random variables in ***X***, and $Pa_{G}(X_{i})$ as the set of parents of $X_{i}\in\boldsymbol{X}$ given DAG *G(****X****,****E****).*

**Property 1:** *P(****X****)* can be factorized over some G as

$P\left( \boldsymbol{X} \right)=\prod_{i=1}^{p} P(X_{i}|Pa_{G}\left( X_{i} \right))$ (1)

Now, we can define Bayesian network (BN) as follows:

**Definition 3:** The pair *(G,P)* is defined as a Bayesian network if *P* factorizes over *G*.

**Remark 1:** The factorization allowed the network to be locally trained, e.g. each $P(X_{i}|Pa_{G}\left( X_{i} \right))$ can be trained independently, which saves a lot of computational time.

Unfortunately, the factorization is not uniquely defined, that is, for some *P* there exists at least two DAGs $G_{1}$ and $G_{2}$ that *P* factorizes over $G_{1}$ and $G_{2}$.

**Definition 4:** ***Q****(P)* defines an equivalent class of *P*, if *P* factorizes over each DAG $G_{i}\in\boldsymbol{Q}$.

In this work, we focusing on estimating any $G\in\boldsymbol{Q}$ instead of estimating every DAGs in $\boldsymbol{Q}$.

Now let us define the conditional dependencies and independencies. We denote $I{nd}_{P}(X_{i};X_{j}|\boldsymbol{Z})$ as $X_{i}$ and $X_{j}$ are conditionally independent given $\boldsymbol{Z}\subset\boldsymbol{X}$ with respect to *P(****X****)*, and $Dep_{P}(X_{i};X_{j}|\boldsymbol{Z})$ as $X_{i}$ and $X_{j}$ are conditionally dependent given $\boldsymbol{Z}$ with respect to $P(\boldsymbol{X})$.

**Definition 5**:

$Ind_{P}\left( X_{i};X_{j} \right|\boldsymbol{Z})\Leftrightarrow P\left( X_{i};X_{j} | \boldsymbol{Z} \right)=P\left( X_{i} | \boldsymbol{Z} \right)P(X_{j}|\boldsymbol{Z})$ (2)

$Dep_{P}\left( X_{i};X_{j} \right|\boldsymbol{Z})\Leftrightarrow P\left( X_{i};X_{j} | \boldsymbol{Z} \right)\neq P\left( X_{i} | \boldsymbol{Z} \right)P(X_{j}|\boldsymbol{Z})$ (3)

One of the most important assumptions we need to include is the faithfulness. To define the faithfulness, let us first define trail:

**Definition 6:** A set of nodes $X_{i_{1}},\ldots,X_{i_{k}}\in\boldsymbol{X}$ forms a trail in the graph $G(\boldsymbol{X},\boldsymbol{E})$ if for every $X_{i_{j}}$ and $X_{i_{j+1}}$, either $X_{i_{j}}\to X_{i_{j+1}}\in\boldsymbol{E}$ or $X_{i_{j}}\leftarrow X_{i_{j+1}}\in\boldsymbol{E}$.

Before we can define an active trail, let us first define descendant. If $X_{j}$ is a descendant of $X_{i}$ then there is a directed path from $X_{i}$ and $X_{j}$.

**Definition 7:** Let $G\left( \boldsymbol{X},\boldsymbol{E} \right)$ be a BN structure, $X_{i_{1}},\ldots,X_{i_{k}}$forms a trail in $G$ and $\boldsymbol{Z}\subset\boldsymbol{X}$. The trail $X_{i_{1}},\ldots,X_{i_{k}}$ is active given $\boldsymbol{Z}$ if

• whenever there is a v-structure: $X_{i_{j}}\to X_{i_{j+1}}\leftarrow X_{i_{j+2}}$, then $X_{i_{j+1}}\in\boldsymbol{Z}$ or a descendant of $X_{i_{j+1}}$ in $\boldsymbol{Z}$

• other nodes are not in $\boldsymbol{Z}$

We need one last definition, d-separation, before we can define faithfulness.

**Definition 8:** In graph $G(\boldsymbol{X},\boldsymbol{E})$, for $X_{i}, X_{j}\in\boldsymbol{X}$ and $\boldsymbol{Z}\subset\boldsymbol{X}$, we say $X_{i}$ and $X_{j}$ are d-separated by $\boldsymbol{Z}$, denoted as$Dsep_{G}(X_{i};X_{j}|\boldsymbol{Z})$, if none of the trails between $X_{i}$ and $X_{j}$ is active given $\boldsymbol{Z}$.

Now let us give the definition on the faithfulness,

**Definition 9:** $P(\boldsymbol{X})$ is faithful to $G\left( \boldsymbol{X},\boldsymbol{E} \right)$ if for any $\boldsymbol{Z\in X}$**:**

$I{nd}_{P}\left( X_{i};X_{j} | \boldsymbol{Z} \right)\Leftrightarrow{Dsep}_{G}(X_{i};X_{j}|\boldsymbol{Z})$ (4)

Under the faithfulness assumption, the terms conditionally independence and d-separation are equivalent; thus, they were used interchangeably through the article.

**Definition 10** A score function $S(G)$ is said to have score-equivalent property if $S(G)$ do not distinguish among equivalent networks. That is, two Bayesian networks, $G_{1}$ and $G_{2}$, are equivalent if and only if $S\left( G_{1} \right)=S\left( G_{2} \right)$.

**Proof of Theorem 1**

Assume $\left( G\left( X,E \right),P\left( X \right) \right)$ is a Bayesian network, $X_{i}\to X_{j}\in E$ and $X^{c}=X\setminus\left\{ X_{i},X_{j} \right\}$. Since $P\left( X \right)$ factorizes on $G\left( X,E \right)$, $X_{i}\to X_{j}\in E$ implies $Dep\left( X_{I};X_{j} \right)$ and $Dep\left( X_{i};X_{j} | X^{c} \right)$. Now let us assume there is an $X_{k}\in X^{c}$ such that $Ind\left( X_{I};X_{j} | X_{k} \right)$. Therefore, all path between $X_{I}$ and $X_{j}$ are blocked by $X_{k}$. Since $X_{k}\in X^{c}$, all path between $X_{i}$ and $X_{j}$ are also blocked by $X^{c}$; thus, $Ind\left( X_{I};X_{j} | X^{c} \right)$ which contradicts with the assumption. Hence, if $Dep\left( X_{I};X_{j} | X^{c} \right)$, then for any $X_{k}\in X^{c}$ we have $Dep\left( X_{I};X_{j} | X_{k} \right)$. Along with$Dep\left( X_{i};X_{j} \right)$, we conclude $X_{i}\in nbr^{C}\left( X_{j} \right)$ and $X_{j}\in nbr^{C}\left( X_{I} \right)$ for any edge in $E$.

**Remark 2:** $n{br}^{C}(X_{i})$ *can be written as*

$$nbr^{C}\left( X_{i} \right)=\left\{ X_{j} \right|dep\left( X_{i};X_{j} \right) \mathrm{and}dep\left( X_{i};X_{j} | X_{k} \right) for any k\neq i,j\}$$

In practice, a symmetric correction may be used, where if $X_{j}\in nbr^{C}\left( X_{i} \right),$but $X_{i}\notin nbr^{C}\left( X_{j} \right),$ then $n{br}^{C}\left( X_{i} \right)={nbr}^{C}\left( X_{i} \right)\setminus\left\{ X_{j} \right\}.$


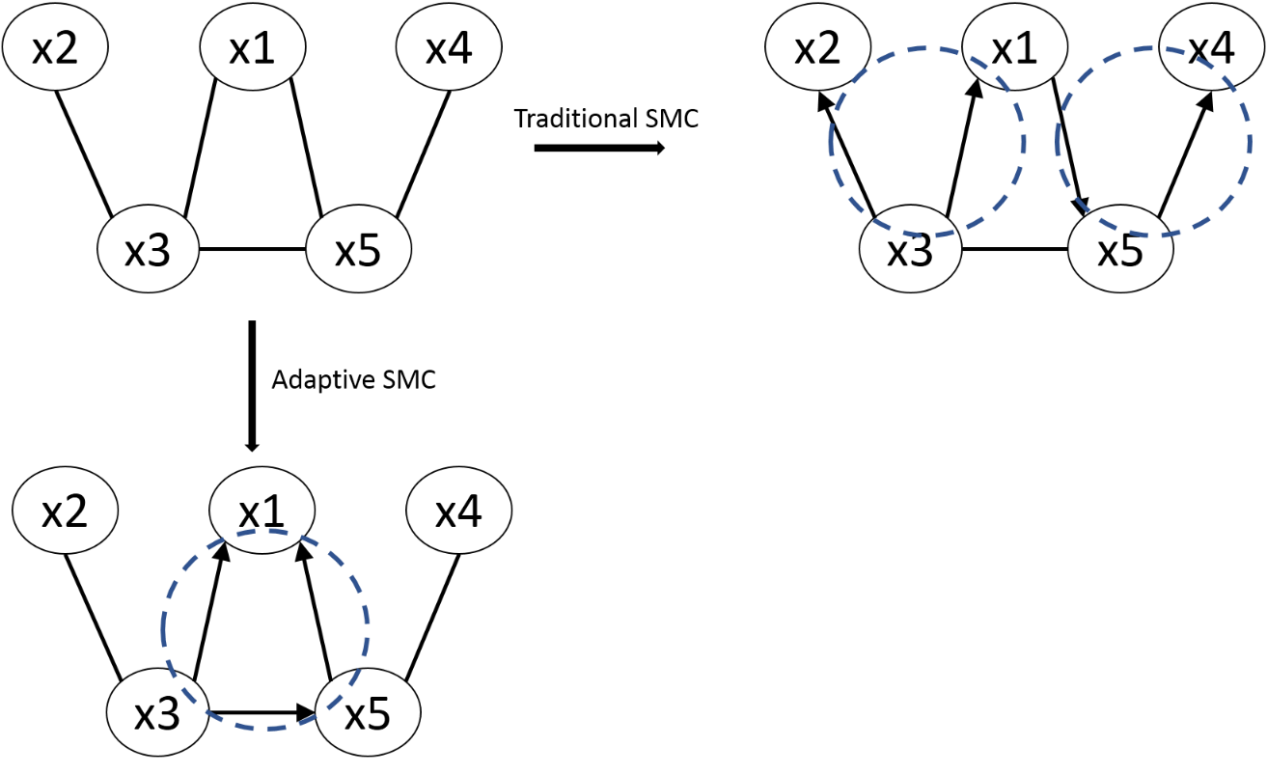


**Figure S1**: Traditional SMC and adaptive SMC. This shows a local skeleton in some step of SMC. In a traditional SMC, it is likely the update sequence set to be $\left( x_{1},x_{2},x_{3} | X \right)\to\left( x_{1},x_{4},x_{5} | X \right)\to\cdots$, while this might limit the later update $\left( x_{3}, x_{5} | X \right)$ to only 2 configurations instead of 3. In adSMC, by selecting sequence based on current structure, we will sample $(x_{1}, x_{3}, x_{5}|X)$ first, which gives us more options on the local sctructure for $(x_{1}, x_{3}, x_{5}|X)$, and this configuration will not affect the options for $(x_{2}, x_{3}|X)$ or $(x_{4}, x_{5}|X)$; thus, increased the diversity of samples.


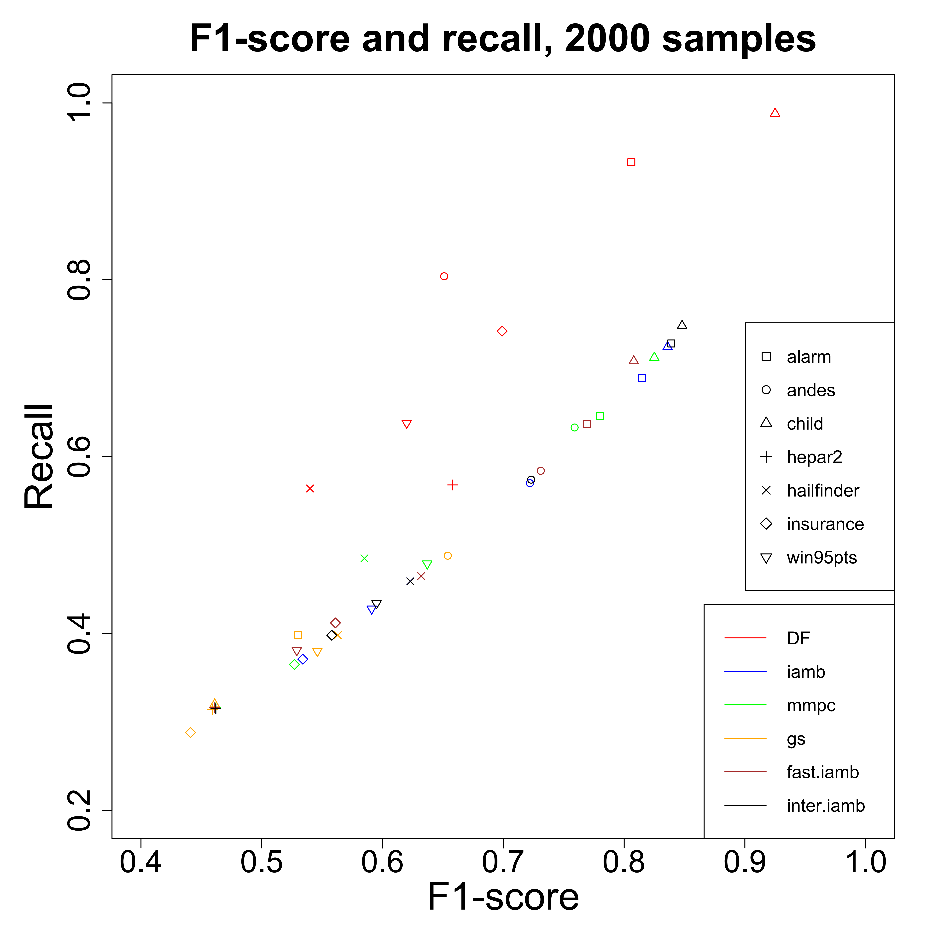

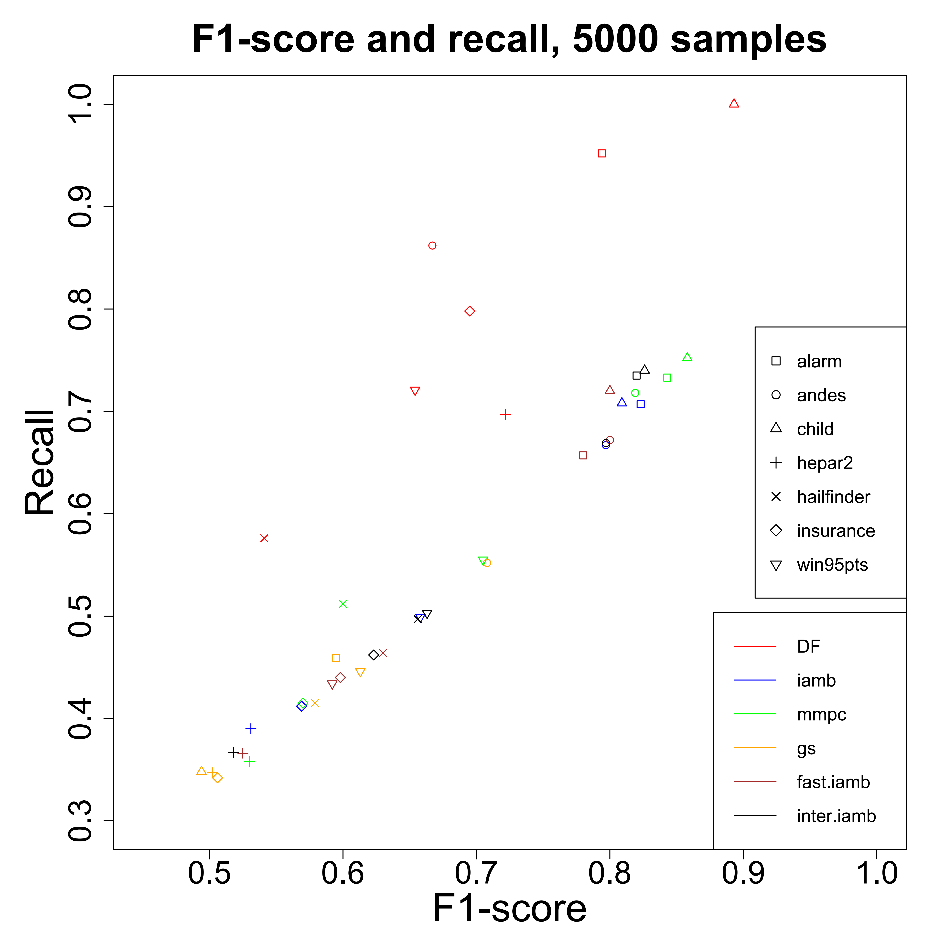


**Figure S2**: F1-score and recall over different methods with observation size 2000 and 5000.


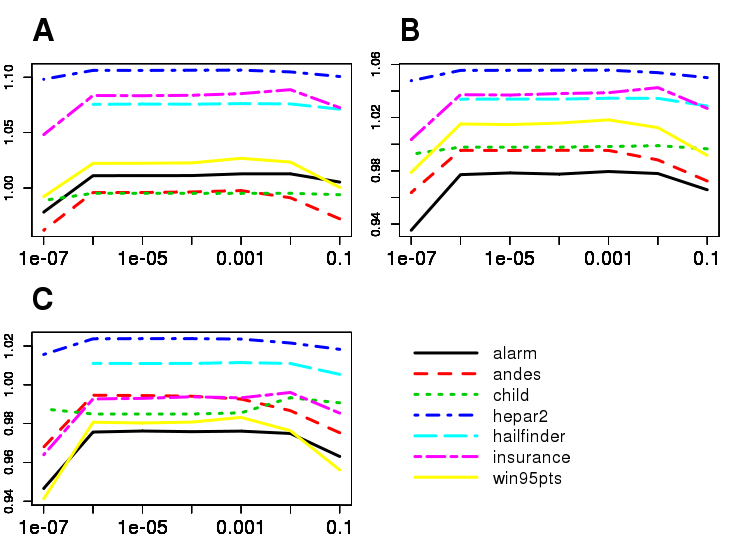


**Figure S3**: The trend of the BIC scores of the learned networks as temperature increases. Observation sizes: (A) 1000, (B) 2000, (C) 5000


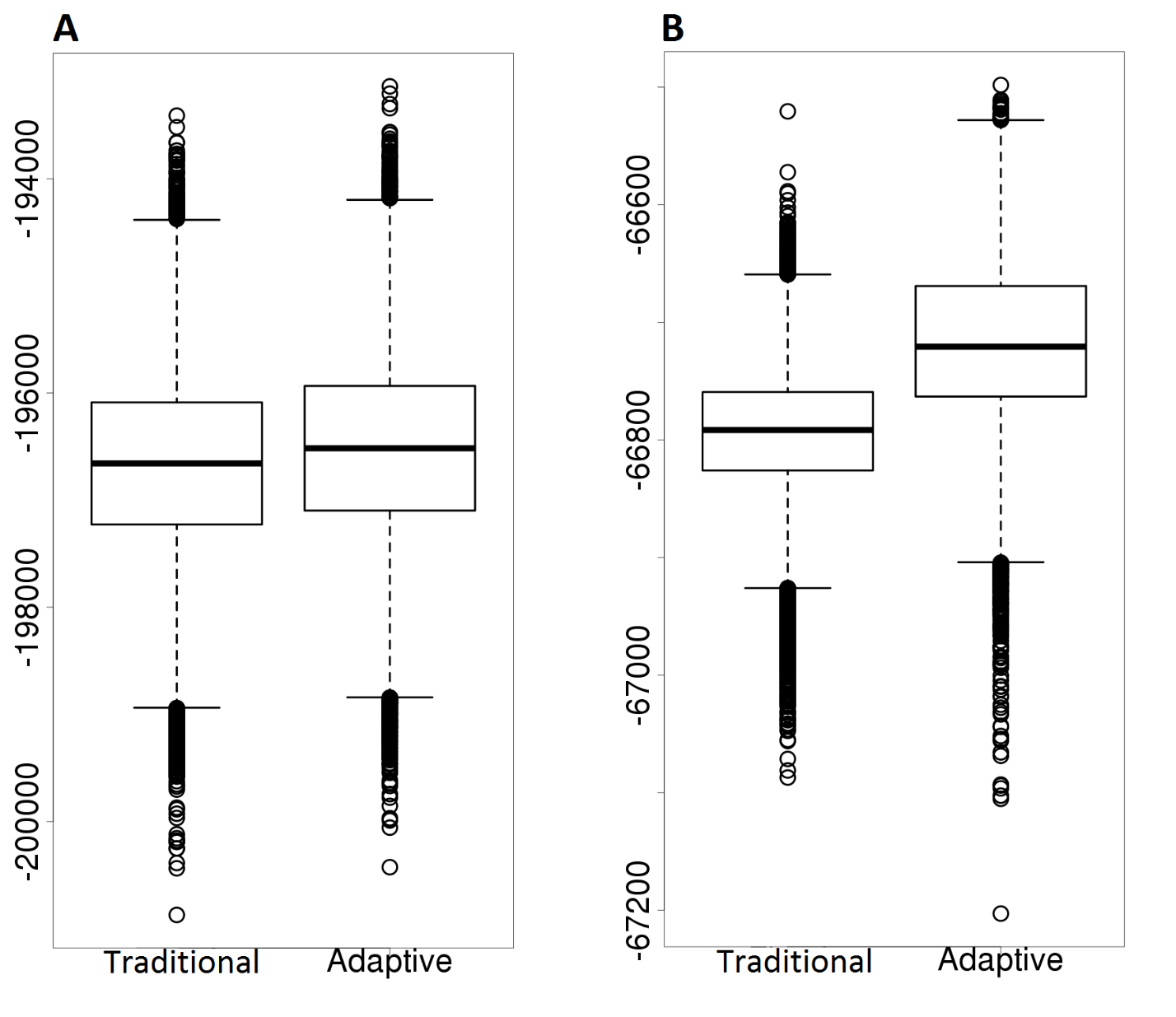


**Figure S4:** The comparison of adSMC and traditional SMC. (A) Andes, (B) hepar2


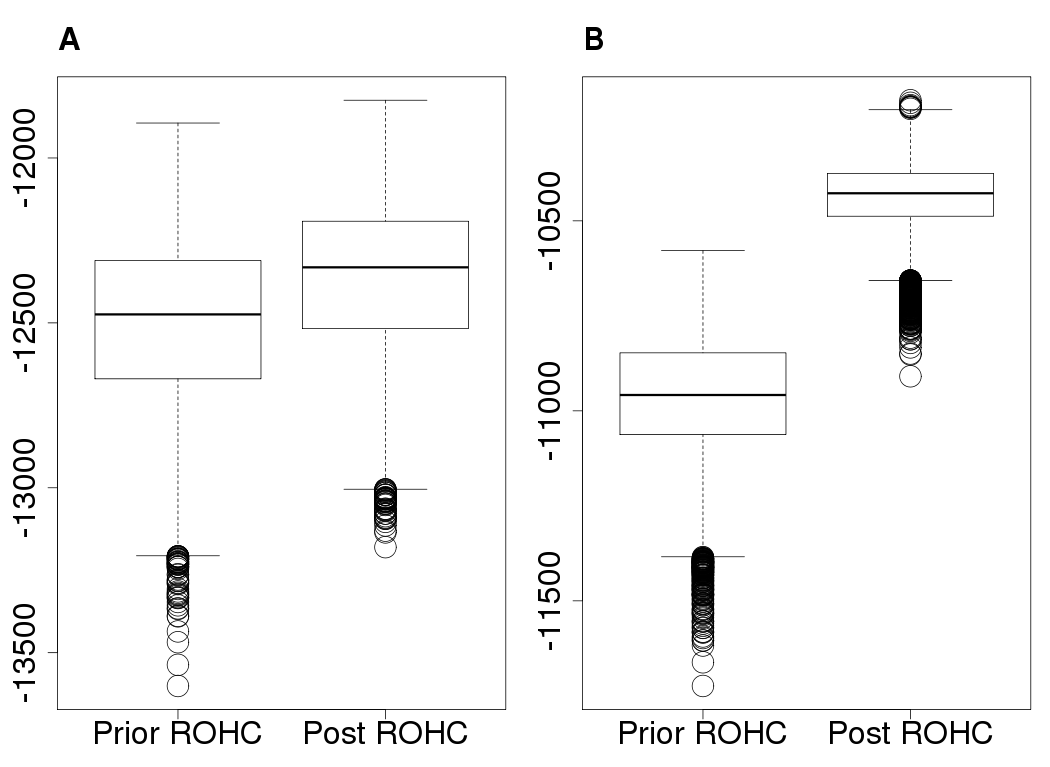


**Figure S5**: The empirical distribution of BIC scores of all SMC samples. (A) alarm network, (B) win95pts network


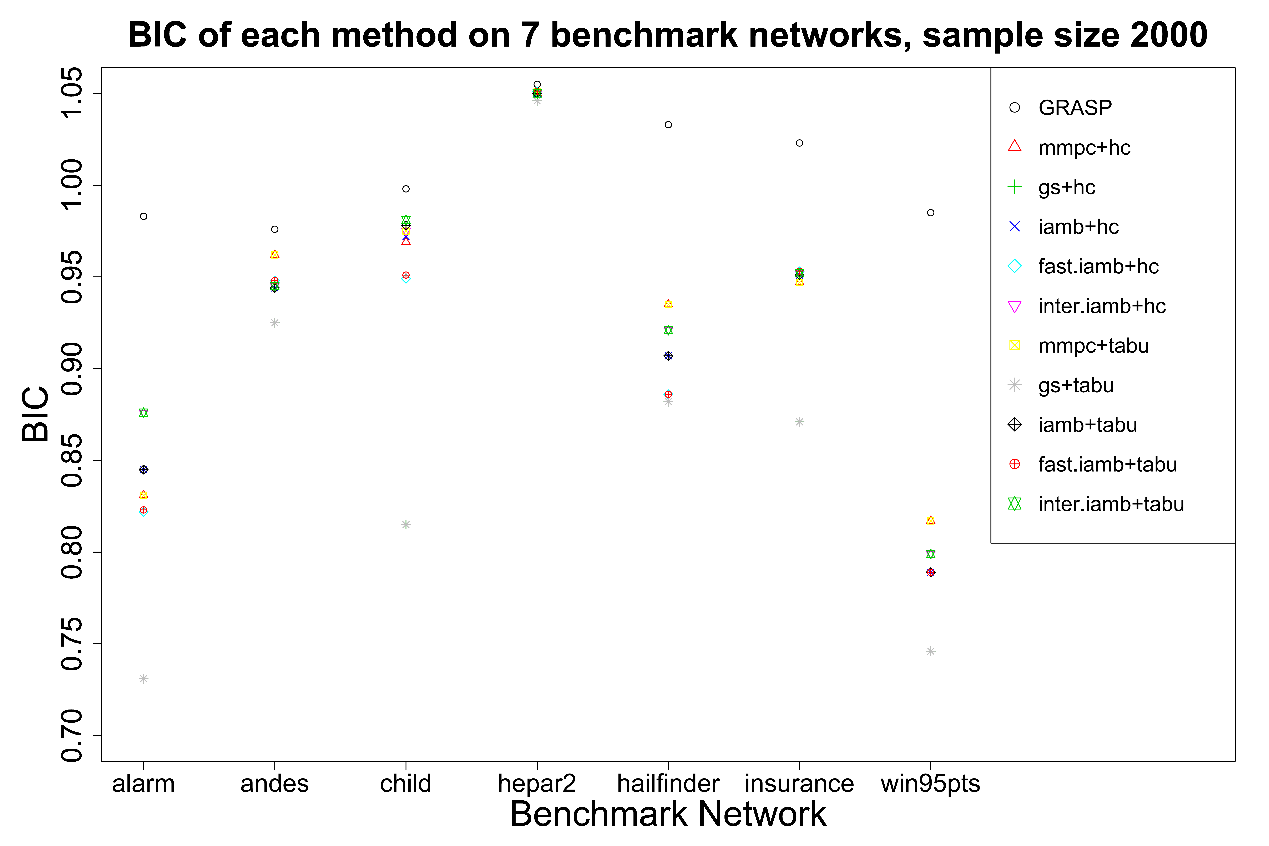


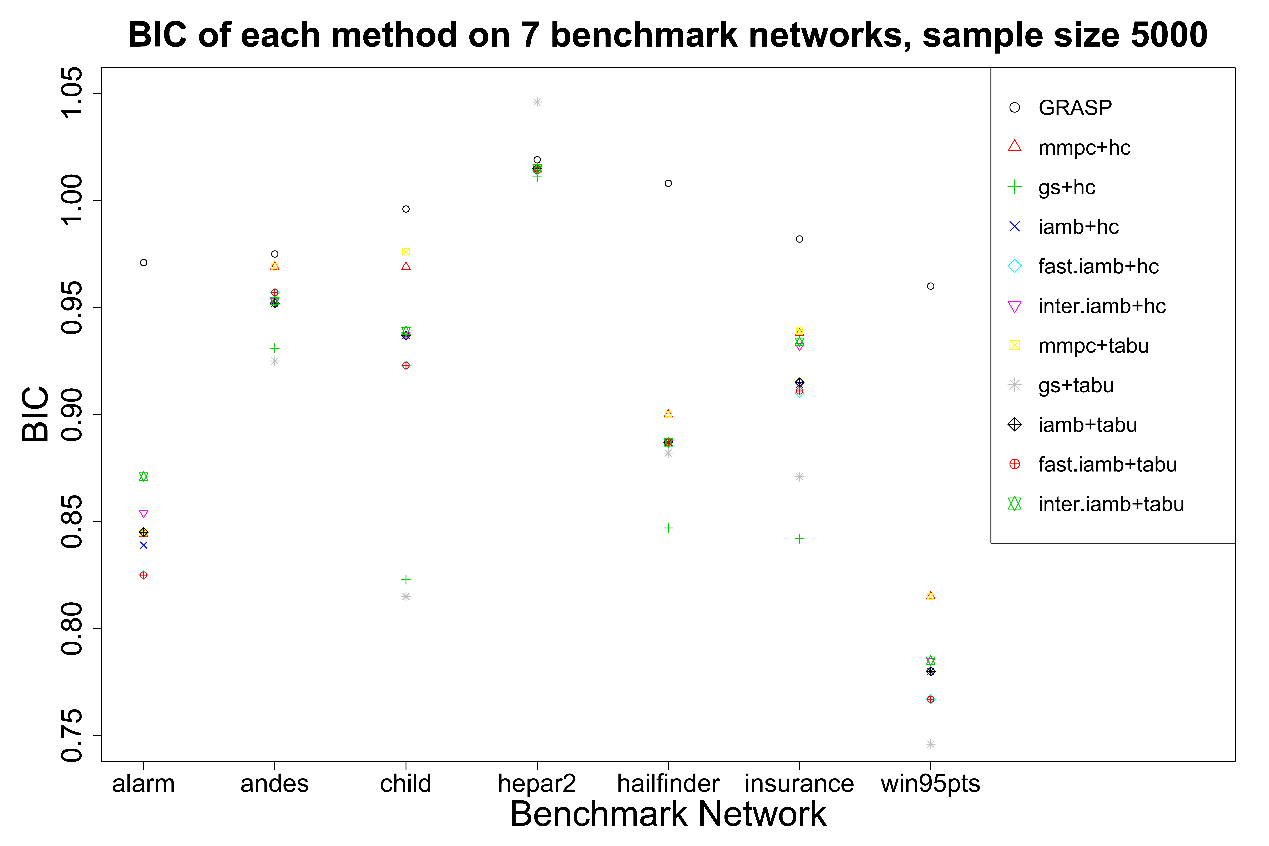


**Figure S6**: BIC scores of all methods on 7 benchmark networks with observation size 2000 and 5000.


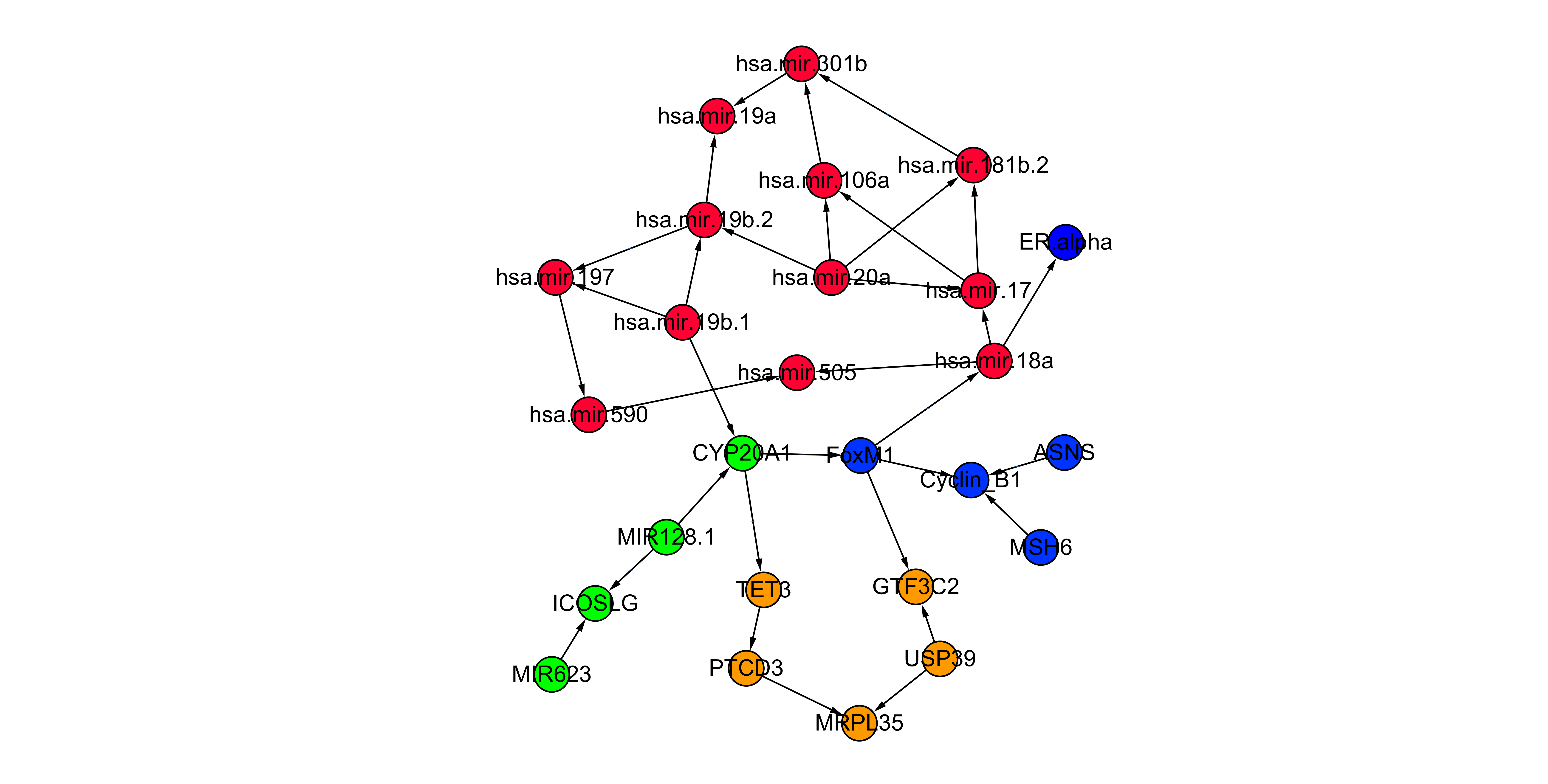


**Figure S7**: The BN structure learned by bnlearn using multiple different genomic features which are highly correlated with the expression of LOC90784. Orange nodes: mRNA transcripts; Red nodes: microRNAs; Blue nodes: protein expressions; Green nodes: DNA methylations.
